# Mindfulness Level Influences the Frequency, Amplitude and Duration of Awake Bruxism Episodes During Standardised Mental Capacity Tasks

**DOI:** 10.1111/joor.70119

**Published:** 2025-11-17

**Authors:** Rafael Chadud Matoso‐Filho, Caio Sberni Pinheiro Souza, Nykolas Jorge Silva Castaldi, Melissa Oliveira Melchior, Fabiane Carneiro Lopes‐Olhê, Simone Cecílio Hallak Regalo, Laís Valencise Magri, Jardel Francisco Mazzi‐Chaves

**Affiliations:** ^1^ Department of Restorative Dentistry, School of Dentistry of Ribeirão Preto University of São Paulo São Paulo Brazil; ^2^ Department of Morphology, Stomatology and Physiology, School of Dentistry of Ribeirão Preto University of São Paulo São Paulo Brazil

**Keywords:** awake bruxism, cognitive tasks, five facet mindfulness questionnaire, mindfulness, surface electromyography

## Abstract

**Background:**

Awake bruxism (AB) is characterised by repetitive or sustained masticatory muscle activity during wakefulness, including clenching, grinding, or mandibular bracing. Recent consensus defines AB as a motor behaviour influenced by psychological and contextual factors. Among these, dispositional mindfulness may play a modulatory role in the frequency and intensity of AB episodes.

**Objective:**

This study aimed to evaluate how standardised cognitive tasks of varying complexity modulate awake bruxism (AB) and to investigate whether dispositional mindfulness is associated with the frequency, duration, and amplitude of AB episodes measured by surface electromyography.

**Methods:**

A cross‐sectional study was conducted with 68 dental students (18–40 years) from the School of Dentistry of Ribeirão Preto, University of São Paulo. Participants were classified into AB and control groups based on self‐report and Ecological Momentary Assessment (EMA). Surface electromyography (sEMG) of the masseter muscle was recorded during five conditions: rest, maximal voluntary clenching, self‐report questionnaires, mathematical tasks of different complexity, and an electronic memory game (Genius). Episodes were analysed at thresholds of 10%, 20%, and 30% of maximal voluntary contraction (Ap10, Ap20, Ap30), extracting frequency, duration, and amplitude. Dispositional mindfulness was assessed using the Five Facet Mindfulness Questionnaire (FFMQ). Statistical analyses included Friedman tests, Mann–Whitney *U* tests, Generalised Estimating Equations (GEE), and Spearman correlations.

**Results:**

Controls showed increased episode frequency at lower thresholds (Ap10) as task complexity rose, whereas AB individuals maintained elevated frequencies across all tasks. Significant between‐group differences were observed at higher thresholds (Ap20, Ap30). Duration and amplitude showed no significant differences, except for a trend of greater amplitude in controls during the Genius task. Within the AB group, higher dispositional mindfulness was associated with fewer and shorter contraction episodes, a pattern consistent with previous research indicating a potential protective association. (notably at Ap30) and shorter durations across tasks, while no associations were observed in controls.

**Conclusion:**

AB individuals exhibited a stable and persistent pattern of masticatory muscle activity, contrasting with the adaptive modulation observed in controls. Higher dispositional mindfulness was linked to fewer and shorter contraction episodes in the AB group, suggesting a protective role. These findings highlight the relevance of integrating psychological traits, such as mindfulness, into the behavioural understanding and management of AB.

## Graphical abstract

Graphical representation of the study evaluating the influence of dispositional mindfulness on the frequency, amplitude, and duration of awake bruxism (AB) episodes during standardised mental capacity tasks. Sixty‐eight adult dental students underwent assessment with the BruxScreen questionnaire, the Five Facet Mindfulness Questionnaire (FFMQ), ecological momentary assessment (BruxApp), and surface electromyography during cognitive tasks of increasing complexity. AB individuals exhibited persistently elevated masticatory muscle activity across tasks, whereas controls showed task‐dependent increases. Higher dispositional mindfulness was associated with fewer and shorter AB episodes—especially under high cognitive demand—highlighting mindfulness as a protective psychological factor in the modulation and management of awake bruxism.

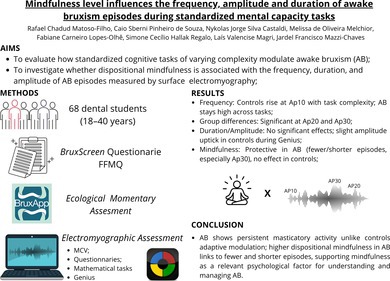

## 1 Introduction

Bruxism is currently defined as a masticatory muscle activity characterised by repetitive or sustained tooth contact and/or mandibular bracing or thrusting movements [1]. This clinical manifestation is classified into two types: sleep bruxism (SB) and awake bruxism (AB). SB refers to the involuntary contraction of the masticatory muscles during sleep, which may present as rhythmic or tonic activity, whereas AB occurs in the awake state, with preserved consciousness, and may manifest as clenching, grinding, bracing, or thrusting. Specifically, clenching and sustained bracing patterns have been associated with muscle fatigue and pain [1, 2, 3].

In recent years, the diagnosis and assessment of bruxism have undergone important conceptual updates [1, 3]. The most recent international consensus recommends that AB should be understood as a motor behaviour rather than a disorder. Its investigation should integrate different assessment modalities: subject‐based (self‐report), clinically based (clinical examination), and device‐based (instrumental approaches), such as surface electromyography (sEMG). This technique plays a relevant role in objectively measuring the electrical activity of masticatory muscles, particularly the masseter and temporalis, although universally accepted parameters for frequency, intensity, and amplitude of episodes are still lacking [1, 3]. In children, research highlights the lack of consolidated management guidelines, while suggesting that non‐pharmacological strategies such as sleep hygiene and mindfulness‐based relaxation may offer safe alternatives [4]. Moreover, the latest proposals for bruxism management emphasise a shift toward personalised and evidence‐based interventions [5].

A recently published analysis examined the diagnosis of AB in light of these new definitions, underscoring the challenges in quantifying its occurrence and the resulting clinical implications [6]. The study emphasised the need for objective criteria and clear guidelines to standardise assessment, particularly during cognitive tasks requiring high levels of concentration, in which AB tends to be more prevalent [6].

The presence of AB in contexts of sustained attention, stressful situations, and activities requiring fine motor coordination has been reported in several international investigations [7, 8, 9]. Cognitive and emotional overload increases the likelihood of tooth clenching episodes, reinforcing the association of AB with psychological and contextual factors [7, 8, 9]. Moreover, activities such as reading, computer use, and mobile device use have been associated with higher AB occurrence and masticatory muscle pain [10, 11, 12, 13].

Overall, the literature indicates that AB is related to multiple factors, including stress, attention, concentration, and psychomotor demands [6, 8, 13]. Recent advancements in understanding AB aetiology further underscore the complex interplay of these factors [14]. These findings suggest that the repetitive motor activity observed in AB may share neurocognitive mechanisms similar to those of other conditions modulated by mindfulness and emotion regulation strategies [15]. Additional evidence shows that mindfulness promotes detachment from negative affective evaluations and fosters the reinterpretation of bodily experiences as transient and non‐threatening sensations, a mechanism associated with symptom reduction in chronic pain conditions [16].

In this context, mindfulness has been considered a relevant resource. It refers to a state of intentional, non‐judgmental awareness in which individuals direct their attention to the present moment, observing thoughts, feelings, bodily sensations, and the environment in a curious and receptive manner. This construct is multifaceted and involves cognitive, emotional, and behavioural components [9, 17, 18, 19, 20]. Recent clinical findings also indicate that mindfulness practice, alone or combined with sleep hygiene measures, is associated with improved sleep quality and reduced bruxism episodes in children [4].

Mindfulness practice enhances self‐awareness and acceptance of internal experiences, potentially reducing stress, improving emotional well‐being, and fostering greater mental clarity [19, 20, 21]. Additional evidence indicates that mindfulness influences attentional processing patterns similar to those involved in AB, particularly under conditions of prolonged mental focus [15, 16, 22]. Functional neuroimaging studies have demonstrated that mindfulness reduces the activation of nociceptive and affective pain‐related circuits in a manner distinct from placebo, reinforcing its specific role in cognitive‐emotional modulation [15].

The assessment of mindfulness can be performed using the Five Facet Mindfulness Questionnaire (FFMQ), which measures five core dimensions: observing, describing, acting with awareness, non‐judging, and non‐reactivity [16, 23, 24, 25, 26]. Recent studies indicate that higher levels of mindfulness are associated with lower AB frequency, better emotional regulation, and reduced prevalence of automatic behaviours [8].

Understanding the interaction between AB, attentional factors, and dispositional traits such as mindfulness may expand the scientific basis for diagnosis, management, and the development of intervention strategies, considering not only the physiological aspects but also the behavioural and emotional dimensions of this condition. Therefore, the present investigation aims to fill an important gap in the literature by exploring, in an integrated manner, the association between cognitive tasks of varying complexity and mindfulness levels with the electromyographic parameters of frequency, intensity, and amplitude of AB. Based on this framework, two null hypotheses were formulated: (H0_1_) during the performance of standardised mental capacity tasks, there are no increases in the frequency, amplitude, or duration of AB episodes; and (H0_2_) higher levels of mindfulness are associated with lower frequency, amplitude, and duration of AB episodes.

## 2 Material and Methods

### 2.1 Study Design and Procedures

This study is a cross‐sectional study.

### 2.2 Ethical Considerations

This study was approved by the Ethics Committee in Research of the Ribeirão Preto School of Dentistry, University of São Paulo (FORP/USP), Brazil (CAAE: 76595123.3.0000.5419). All participants received detailed information about the study and signed the informed consent form prior to enrollment. The research protocol complied with the principles of the Declaration of Helsinki and followed the Brazilian National Health Council Resolution 466/12.

### 2.3 Sample

Participants were undergraduate dental students from FORP/USP, of both sexes, aged over 18 years and enrolled between the first and fourth years of the program. Sample size calculation was performed using Bioestat 6.0 software, based on the estimated prevalence of awake bruxism (15.6%–48%) [27, 28]. The parameters considered were: statistical power of 0.80, significance level of 0.05, and effect size of 0.40 (Cohen's test for one‐way ANOVA, between‐subjects design). The final sample comprised 68 healthy young adults aged 18–40 years, divided into two groups: (1) awake bruxism (AB) and (2) control. Diagnosis of AB followed the most recent international consensus criteria [1, 2], requiring a positive self‐report (‘regularly, frequently or always’) for at least 60% of the Bruxscreen‐inspired questions [29]. These participants who indicated ‘regularly, frequently or always’ for at least 60% of the Bruxscreen questions were invited to participate in an Ecological Momentary Assessment (EMA) using the mobile application *BruxApp* for one week, to confirm the diagnostic [30].

The dental student sample was selected for its specific relevance: they experience high cognitive demands and psychosocial stress, factors known to influence awake bruxism. This population, also investigated in previous studies [31, 32, 33] serves as an ideal model for investigating the interaction between cognitive load, mindfulness, and bruxism, despite limitations in generalizability to the broader adult population.

Exclusion criteria included: history of neurological or psychiatric disorders, presence of chronic orofacial pain, current use of medication influencing muscle activity, botulinum toxin injection in the masticatory muscles in the past 6 months, undergoing orofacial myofunctional therapy in the previous 12 months, or communication difficulties precluding data collection.

#### 2.3.1 Phase 1—Assessment of AB and Ecological Momentary Assessment (EMA)

The presence of AB was determined using self‐report questions adapted from the *BruxScreen* questionnaire [34]. Items covered both bruxism‐related behaviours (e.g., tooth clenching, grinding, mandibular bracing) and mandibular symptoms (e.g., pain, tension, fatigue). Questions were answered on a five‐point frequency scale (never, occasionally, regularly, frequently, always). For classification as AB, participants had to endorse ‘regularly,’ ‘frequently,’ or ‘always’ in ≥ 60% of items.

To confirm the diagnostic as previously described, eligibility for participation in the Ecological Momentary Assessment (EMA) required that participants reported the frequencies regularly, frequently, or always in at least 60% of the BruxScreen‐inspired questions, corresponding to at least 4 out of the 7 items.

The smartphone application BruxApp was employed, which described five oral conditions related to awake bruxism behaviours. Participants were instructed to select one of the options whenever prompted by the application alarm. The oral conditions included in the application were: relaxed masticatory muscles, mandibular bracing (masticatory muscle contraction without dental contact), teeth in contact, teeth clenching, and teeth grinding.

The testing period for the EMA lasted one week. Participants were instructed to program 12 alarms per day during waking hours, which represented the minimum number required to ensure a valid daily assessment of awake bruxism frequency, according to a previous study [30]. Each time the alarm was triggered, participants were required to select one of the five conditions that best described their current oral behaviour status.

It is important to emphasise that participants did not receive any instructions regarding the control of oral behaviours related to awake bruxism at the baseline assessment. Therefore, no Ecological Momentary Intervention was conducted through the application, and no reminders were programmed to raise awareness or promote control of such behaviours. Only the EMA was carried out during this one‐week period. Daily reports were automatically sent to the researchers, who calculated the daily frequency of awake bruxism episodes, expressed as a percentage.

#### 2.3.2 Phase 2—Mindfulness Levels

Mindfulness was assessed using the Five Facet Mindfulness Questionnaire (FFMQ), validated for the Brazilian population [6, 23, 24, 25, 35]. This 39‐item self‐report scale, answered on a 5‐point Likert scale, evaluates five dimensions: observing, describing, acting with awareness, non‐judging of inner experience, and non‐reactivity to inner experience. Higher scores indicate higher levels of mindfulness.

#### 2.3.3 Phase 3—Electromyographic Assessment

Surface electromyography (sEMG; Noraxon, USA) was employed to record bilateral masseter activity under standardised laboratory conditions. Participants were seated in an upright position without head support, feet resting flat on the floor, and were instructed to minimise head movements, refrain from speaking, and avoid touching the electrodes during recordings. A quiet, temperature‐controlled room was used to reduce environmental interference, in line with recent methodological recommendations for awake bruxism (AB) assessment [36, 37].

During testing disposable silver/silver chloride bipolar electrodes, with a diameter of 10 mm and an interelectrode distance of 20 mm (Duo‐Trode; Myo‐Tronics Inc., Seattle, WA, USA) were placed over the right masseter muscle following the SENIAM (Surface EMG for Non‐Invasive Assessment of Muscles) guidelines, with careful skin preparation to reduce impedance (< 5 kΩ). A reference electrode was positioned over the styloid process. The electrode placement protocol was consistent with prior EMG studies investigating diurnal jaw motor activity [36, 37, 38].

### 2.4 Recording Protocol

EMG activity was continuously recorded during four experimental conditions:

1. Resting state (5 min)—baseline muscle activity with relaxed jaw posture.
2. MVC trials—three repetitions of 5‐s maximal clenching in maximum intercuspation.
3. Self‐administered questionnaires (BruxScreen and FFMQ; ~10 min).
4. Mathematical tasks (low, moderate, and high complexity; ~10 min each), adapted from Macedo, Petty, Passos (2000) [39] and Lamas, Oliveira, Amorim (2022) [40].
5. Cognitive game task (Genius electronic sequence memory game; 10 min), engaging fine motor control and divided attention.

These tasks were selected to simulate varying levels of cognitive and attentional demand, reflecting the multidimensional assessment framework proposed for AB [6, 36].

Similar task‐based EMG paradigms have been shown to elicit distinct muscle activation patterns, supporting their use in experimental bruxism studies [36].

### 2.5 Experimental Tasks

Participants performed the following tasks, during which EMG signals were continuously recorded:

1. Resting condition.
2. Maximal voluntary clenching in intercuspation (3 × 5 s).
3. Completion of self‐report questionnaires (BruxScreen and FFMQ)—10 min.
4. Mental arithmetic tasks (10 min) with three complexity levels (low, medium, high), adapted from previous protocols [39, 40]. Calculations involved dice rolls and sequential multiplications with escalating complexity, performed without calculators.
5. Electronic game (Genius—colour sequence task)—10 min, designed to elicit cognitive load, stress, and fine motor coordination.

Raw EMG signals were amplified, filtered, and processed with MyoTrace 400 software (Noraxon, USA). The root mean square (RMS) amplitude (μV) was extracted for analysis.

#### 2.5.1 Signal Calibration and Normalisation

To normalise EMG signals, participants performed three 5‐s maximal voluntary contractions (MVCs) in maximum intercuspation, with 5‐s rest intervals. The mean peak amplitude across trials was defined as 100% MVC. Normalisation procedures are recommended to account for inter‐individual variability and facilitate comparisons across experimental conditions [36, 37].

Contraction episodes were defined using amplitude thresholds of 10% (Ap10), 20% (Ap20), and 30% (Ap30) of MVC, as suggested by prior studies on awake bruxism metrics [6, 38]. For each threshold, three parameters were extracted: frequency (number of episodes), mean duration (s), and mean amplitude (μV). Additionally, the root mean square (RMS) of the EMG signal was calculated for each task to capture global activity levels, a method validated for both short‐term and prolonged recordings [36, 38].

#### 2.5.2 Signal Processing

Raw EMG signals were sampled at 1000 Hz, band‐pass filtered (10–500 Hz), and notch‐filtered at 60 Hz to remove electrical noise. Data were rectified and smoothed using RMS calculations with a 100 ms moving window. Bursts were defined as events exceeding 2× baseline amplitude with a minimum duration of 0.25 s, following operational definitions established in diurnal EMG protocols [37].

All recordings were stored and analysed offline using MyoTrace 400 software (Noraxon, USA). EMG indices were computed per task and normalised to MVC. To account for day‐to‐day variability in EMG waveforms, reliability checks were performed across tasks, in line with recent recommendations emphasising reproducibility in AB assessment [36, 37].

### 2.6 Statistical Analysis

Within‐group comparisons across tasks (AB vs. controls) were conducted using Friedman's test for repeated measures. To interpret directionality, an a priori intra‐subject contrast Δ(*Q*–*G*) was calculated (difference between questionnaire task [low attentional demand] and Genius game [high attentional demand]). Between‐group differences were tested using Mann–Whitney *U*. Data were expressed as medians and interquartile ranges (IQR). Significance was set at *α* = 0.05; 0.05 < *p* < 0.10 was considered a trend.

Associations between dispositional mindfulness (total FFMQ *z*‐scores) and EMG outcomes were tested using Generalised Estimating Equations (GEE) with participant clustering and exchangeable correlation. Poisson family with log link was applied for frequency outcomes, yielding incidence rate ratios (IRR) with 95% CI. Gaussian family with log(*y* + 1) was applied for duration and amplitude, expressed as percentage variation. Complementary Spearman's correlations (*ρ*) explored associations between mindfulness and outcomes by task and group. Analyses were performed in R (v.4.5.1).

## 3 Results

### 3.1 Sample Characterisation

The final sample comprised 68 individuals, with a mean age of 22.5 ± 2.7 years (median = 22.5; interquartile range [IQR] = 21.0–24.0). The sex distribution included 38 men (55.9%) and 30 women (44.1%). Of these, 33 participants (48.5%) were classified as having awake bruxism, while the remaining 35 served as controls.

The awake bruxism group completed an EMA using the BruxApp (1 week), enabling real‐time monitoring of orofacial muscle activity during daily activities. The mean frequency of awake bruxism behaviours was 44.7%. Among the specific behaviours recorded, tooth contact was the most frequent (mean = 14.06 episodes), followed by bracing (mean = 6.55 episodes) and clenching (mean = 4.30 episodes), whereas grinding was rare (mean = 0.52 episodes). These findings indicate that sustained low‐intensity tooth contact represents the predominant awake bruxism manifestation in this sample, reinforcing the value of ecological momentary assessment for accurately characterising real‐world bruxism patterns.

### 3.2 Frequency, Amplitude, and Duration of Masticatory Muscle Contraction Events

Table 1 summarises the frequency of masticatory muscle contraction episodes (Ap10, Ap20, and Ap30 thresholds) during the cognitive tasks of Questionnaire, Mathematics, and Genius, comparing individuals with probable AB and controls.

**TABLE 1:** Frequency of masticatory muscle contraction episodes (Ap10, Ap20, Ap30) during standardised cognitive tasks in individuals with awake bruxism (AB) and controls.

Measure	Group	Questionnaire (*Q*) median [IQR]	Mathematics (*M*) median [IQR]	Genius game (*G*) median [IQR]	Friedman test (*Q* vs. *M* vs. *G*)	*Q*–*G* difference (Median)	Mann–Whitney *U* test (Δ *Q*–*G*; AB × Control)
Ap10	Control	1 [0–2]	2 [1–3]	2 [1–3]	*χ* ^2^ = 4.24; *p* = 0.044	−1	***p* = 0.04**
	AB	2 [1–3]	2 [1–2]	1 [0–2]	*χ* ^2^ = 6.68; *p* = 0.03	0	
Ap20	Controle	1 [0–2]	2 [1–3]	2 [1–3]	*χ* ^2^ = 2.03; *p* = 0.362	0	***p* = 0.001**
	BV	2 [0–2]	3 [1–3]	4 [1–4]	*χ* ^2^ = 7.94; *p* = 0.03	0	
Ap30	Controle	0 [0–1]	1 [0–2]	1 [0–2]	*χ* ^2^ = 1.42; *p* = 0.02	−1	***p* = 0.003**
	BV	2 [0–3]	3[1–2]	3 [0–3]	*χ* ^2^ = 6.42; *p* = 0.001	0	

*Note:* Values are presented as medians [interquartile range, IQR]. The Friedman test was applied for intragroup comparisons (*Q* vs. *M* vs. *G*). The *Q*–*G* difference represents the individual variation between tasks (delta). Mann–Whitney *U* test compared *Q*–*G* differences (Δ *Q*–*G*) in AB and control groups. *p* < 0.05 was considered statistically significant (bold); values between 0.05 and 0.10 were interpreted as trends. Ap10, Ap20, and Ap30 correspond to contraction episodes exceeding 10%, 20%, and 30% of MVC, respectively.

Abbreviations: AB, awake bruxism; *G*, genius; *M*, mathematics; *Q*, questionnaire.

At Ap10, both controls (*χ* ^2^ = 4.24; *p* = 0.044) and AB participants (*χ* ^2^ = 6.68; *p* = 0.03) exhibited significant variation across tasks. In the control group, frequency was low during the Questionnaire (1 [0–2]) and increased during Mathematics and Genius (2 [1–3]), suggesting that higher cognitive demand elevated the occurrence of episodes. In contrast, AB individuals showed higher baseline frequency during the Questionnaire (2 [1–3]) that remained stable across subsequent tasks, reflecting a persistence of the behaviour across conditions. Between‐group comparisons revealed significant differences in the Questionnaire–Genius contrast (*p* = 0.04), indicating that frequency modulation occurred differently between groups.

At Ap20, only AB participants demonstrated significant differences between tasks (*χ* ^2^ = 7.94; *p* = 0.03), whereas the control group remained stable (*p* = 0.362). Intergroup comparisons also showed differences in the *Q*–*G* contrast (*p* = 0.001), suggesting that even at higher contraction thresholds, AB individuals consistently exhibited more frequent episodes across tasks.

At Ap30, the AB group again showed significant variation across tasks (*χ* ^2^ = 6.42; *p* = 0.001), whereas the control group displayed a less pronounced pattern. Between‐group analysis confirmed significant differences in the *Q*–*G* contrast (*p* = 0.003).

Taken together, these findings demonstrate that in controls, the frequency of masticatory muscle contraction episodes is more sensitive to increased cognitive load, intensifying during more demanding tasks. Conversely, in individuals with AB, episodes are already more frequent at baseline (Questionnaire) and remain elevated across subsequent tasks, highlighting a pattern of greater consistency.

**FIGURE 1:**
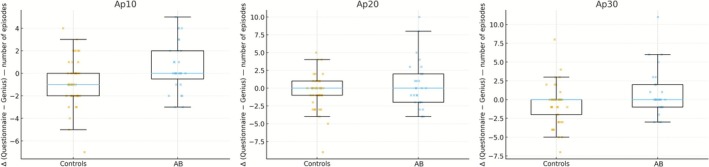
Individual differences Δ(*Q*–*G*) in the frequency of masticatory muscle contraction episodes between the Questionnaire (*Q*) and Genius (*G*) tasks, comparing controls and AB participants at Ap10, Ap20, and Ap30 thresholds. Each panel shows boxplots (median and IQR) with individual data points. Negative Δ (*Q*–*G*) values indicate more episodes during Genius than Questionnaire, whereas positive values indicate the opposite. The *p*‐value of the two‐tailed Mann–Whitney test for between‐group comparison (AB vs. controls) is displayed at the top of each panel. This figure illustrates how task‐related modulation differs between groups across the different intensity thresholds (Ap10/Ap20/Ap30).

Figure 1 depicts the individual variation Δ(*Q*–*G*) (difference between the Questionnaire and Genius tasks) across the three frequency thresholds analysed (Ap10, Ap20, and Ap30), comparing individuals with AB and controls. In the control group, Δ tended to be negative at Ap10, reflecting a higher occurrence of contraction episodes during the Genius task, when cognitive demand increased, compared to the Questionnaire. In contrast, in the AB group, Δ approached zero or was less negative, suggesting persistence of episode frequency even during tasks with lower cognitive demand.

At higher intensity thresholds (Ap20/Ap30), the pattern of between‐group differences remained evident: controls exhibited smaller variations across tasks, whereas AB individuals maintained consistently elevated episode levels, resulting in Δ distributions that differed significantly between groups (Mann–Whitney *p*‐values are annotated in each panel).

Taken together, these findings reinforce that cognitive load primarily increases the frequency of contraction episodes in controls, whereas in AB individuals, occurrence is already higher and remains constant from the baseline Questionnaire task.

### 3.3 Duration and Amplitude of Masseter Muscle Contraction Episodes

Table 2 presents the results for the duration (s) and amplitude (μV) of masticatory muscle contraction episodes during the three standardised cognitive tasks (Questionnaire, Mathematics, and Genius). No significant differences were found between tasks in either group for any of the measures, as indicated by Friedman's test (all *p* > 0.36). The median duration of episodes remained relatively stable across tasks, ranging from 5 to 6 s in controls and from 4 to 5 s in AB individuals. Similarly, amplitudes ranged from 40 to 60 μV in both groups, without significant intragroup differences.

**TABLE 2:** Duration (s) and amplitude (μV) of masticatory muscle contraction episodes during standardised cognitive tasks in individuals with awake bruxism (AB) and controls.

Measure	Group	Questionnaire (*Q*) median [IQR]	Mathematics (*M*) median [IQR]	Genius GAME (*G*) median [IQR]	Friedman test (*Q* vs. *M* vs. *G*)	*Q*–*G* difference (Median)	Mann–Whitney (AB vs. Control)
Duration (s)	Control	5 [4–7]	6 [5–8]	6 [4–7]	*χ* ^2^ = 2.03; *p* = 0.363	−1.0	*p* = 0.673 (*Q*) *p* = 0.624 (*M*) *p* = 0.435 (*G*)
	AB	4 [3–6]	5 [3–6]	4 [3–6]	*χ* ^2^ = 1.01; *p* = 0.603	−1.0	—
Amplitude (μV)	Control	44.6 [36–55]	45.0 [37–56]	50.0 [39–61]	*χ* ^2^ = 2.00; *p* = 0.368	−10.5	*p* = 0.126 (*Q*) *p* = 0.726 (*M*) *p* = 0.250 (*G*)
	AB	50.0 [41–62]	36.0 [30–49]	38.5 [31–50]	*χ* ^2^ = 0.26; *p* = 0.877	0.0	—

*Note:* Values are presented as medians [interquartile range, IQR]. The Friedman test was applied for intragroup comparisons (*Q* vs. *M* vs. *G*). The *Q*–*G* difference represents the individual variation between tasks (delta). The Mann–Whitney *U* test compared AB versus controls within each task. The Δ Amplitude (*Q*–*G*) showed a trend (*p* = 0.058) toward a distinct pattern between groups: controls exhibited relatively higher amplitudes in *G* compared with *Q*, whereas AB individuals maintained stable values.

Abbreviations: AB, awake bruxism; *G*, genius; *M*, mathematics; *Q*, questionnaire.

Between‐group comparisons (Mann–Whitney *U* test) also revealed no statistically significant differences in duration or amplitude for any of the tasks (all *p* > 0.12 for amplitude; all *p* > 0.43 for duration). However, when analysing the contrast between Questionnaire and Genius (Δ*Q*–*G*), a trend was observed for amplitude (*p* = 0.058), suggesting a distinct pattern between groups: while controls exhibited relatively higher amplitudes during Genius compared to Questionnaire (median Δ = −10.5 μV), AB individuals maintained stable values (median Δ = 0).

These findings indicate that, unlike frequency, which was sensitive to group differences, the duration and amplitude of episodes exhibited more homogeneous behaviour, with only a trend toward group interaction for amplitude.

### 3.4 Mindfulness, Masticatory Muscle Contraction, and Awake Bruxism

Table 3 presents the effect of dispositional mindfulness (total FFMQ score) on the frequency (Ap10/Ap20/Ap30), duration, and amplitude of contraction episodes, analysed by task (Questionnaire, Mathematics, Genius) and by group (AB and controls). The FFMQ score was derived from the 39 items with standardised reverse scoring and proration when ≥ 34/39 responses were available, standardised into *z*‐scores. For frequency outcomes, we applied Poisson GEE models (participant as cluster; exchangeable correlation structure), with Genius as the reference and dummy variables for Mathematics and Questionnaire, including FFMQ×task interactions to estimate task‐specific FFMQ effects (reported as IRR per +1 SD increase in FFMQ). For duration and amplitude, Gaussian GEE models were fitted on log(*y* + 1) with the same structure, and effects were expressed as %Δ per +1 SD.

**TABLE 3:** Significant effects of dispositional mindfulness (total FFMQ, per +1 SD) on frequency (IRR) and duration (%Δ) by task (*Q*, *M*, *G*), stratified by group.

Group	Outcome	Task	Effect size (95% CI)	*p*	Parameter
AB	Ap30	Genius (*G*)	IRR 0.52 (0.34 to 0.80)	**0.003**	Frequency
AB	Ap30	Mathematics (*M*)	IRR 0.42 (0.25 to 0.70)	**0.001**	Frequency
AB	Ap20	Genius (*G*)	IRR 0.82 (0.68 to 0.98)	**0.028**	Frequency
AB	Duration	Questionnaire (*Q*)	−25.3% (−38.2% to −9.7%)	**0.003**	%Δ (log)
AB	Duration	Mathematics (*M*)	−24.9% (−37.7% to −9.5%)	**0.003**	%Δ (log)
AB	Duration	Genius (*G*)	−17.3% (−28.9% to −3.9%)	**0.013**	%Δ (log)

*Note:* Interpretation: IRR < 1 indicates fewer episodes per +1 SD increase in FFMQ; negative %Δ indicates a reduction in mean duration (models on log scale). Trends (0.05 ≤ *p* < 0.10): AB–Ap20/Mathematics IRR = 0.74 (0.55–1.01), *p* = 0.061. No significant effects were observed in the Control group (all tasks, all outcomes). Modelling: Frequency (Ap10/Ap20/Ap30) was analysed using Poisson GEE with participant as cluster; task‐specific effects were obtained via linear combinations (FFMQ × *G*, FFMQ × *Q*, FFMQ × *M*). Duration and amplitude were modelled using Gaussian GEE on log(*y* + 1) with the same parametrization; results expressed as %Δ. The bold values indicate statistically significant differences between the groups.

Among AB individuals, higher FFMQ scores were associated with lower frequency of episodes, particularly at higher intensity thresholds. At Ap30, significant negative associations were observed with Genius (IRR = 0.52; 95% CI: 0.34–0.80; *p* = 0.003) and Mathematics (IRR = 0.42; 95% CI: 0.25–0.70; *p* = 0.001), where a +1 SD increase in FFMQ scores was consistently associated with approximately 48% and 58% lower episode frequencies, respectively. At Ap20, a reduction was also found in Genius (IRR = 0.82; 95% CI: 0.68–0.98; *p* = 0.028), with a similar trend in Mathematics (IRR = 0.74; 95% CI: 0.55–1.01; *p* = 0.061). Additionally, in the AB group, higher FFMQ was associated with shorter episode duration across all tasks: Questionnaire −25.3% (95% CI: −38.2% to −9.7%; *p* = 0.003), Mathematics −24.9% (95% CI: −37.7% to −9.5%; *p* = 0.003), and Genius −17.3% (95% CI: −28.9% to −3.9%; *p* = 0.013). No associations were observed with amplitude in any task among AB individuals.

In the control group, no significant effects of FFMQ were detected on frequency, duration, or amplitude during Questionnaire, Mathematics, or Genius tasks. Taken together, these analyses indicate that higher dispositional mindfulness is linked to fewer contraction episodes (especially those of greater intensity) and shorter duration among individuals with AB, including in the Questionnaire task, whereas in controls, mindfulness did not modulate the evaluated outcomes.

In the AB group, negative correlations were observed between FFMQ scores and frequency, particularly at Ap30, with clearer associations in the Mathematics and Genius tasks. These findings indicate that higher levels of mindfulness are linked to fewer intense contraction episodes. Negative associations between FFMQ and duration also emerged across all three tasks (*Q*/*M*/*G*), suggesting shorter episodes among participants with higher mindfulness.

In contrast, correlations between FFMQ and amplitude remained close to zero, showing no consistent pattern. In the control group, correlations were weak or absent across all outcomes and tasks, without any clear organisation.

**FIGURE 2:**
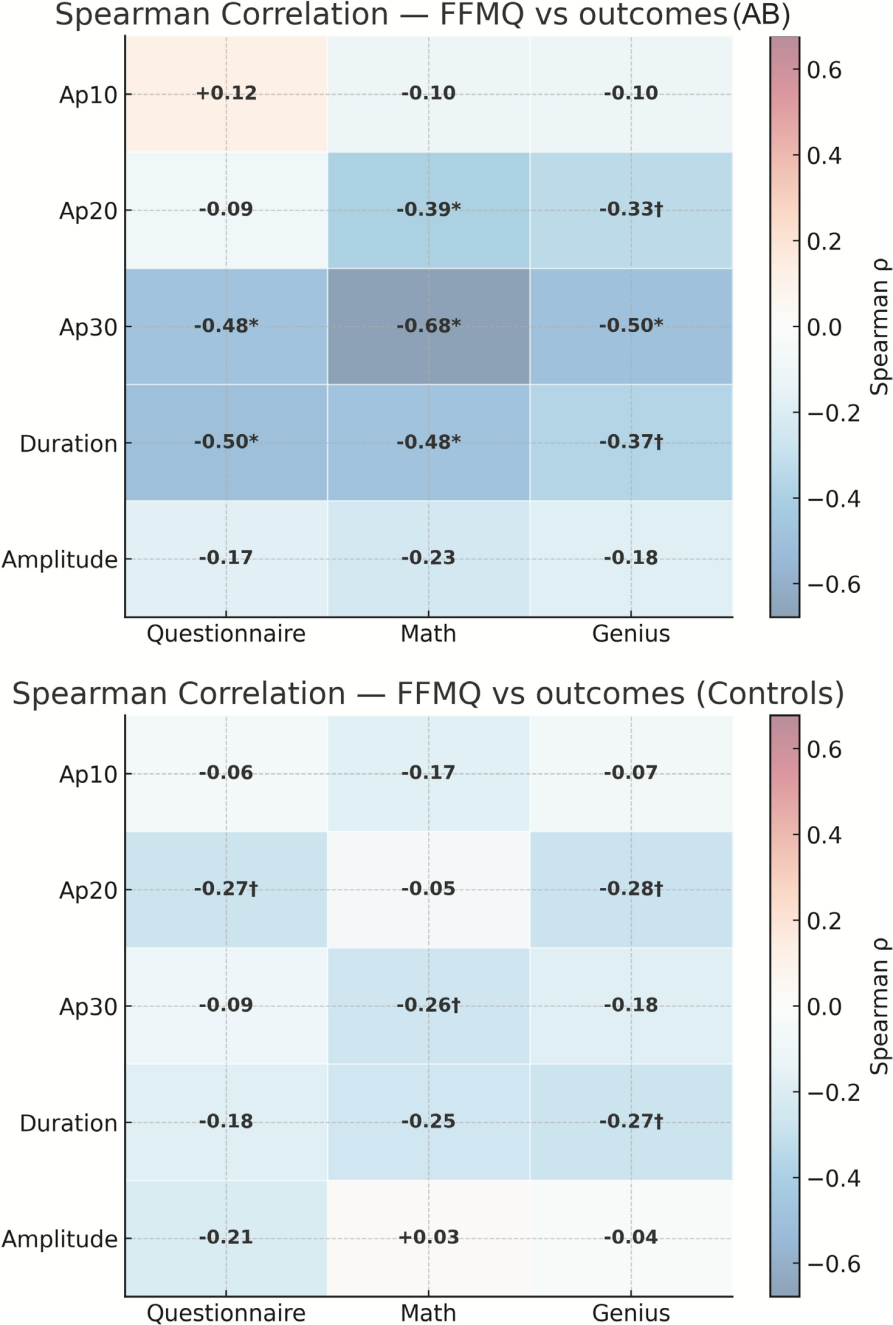
Spearman correlations between dispositional mindfulness (total FFMQ) and masticatory muscle contraction outcomes, by task. Upper panel: probable awake bruxism (AB) group. Lower panel: controls. Columns represent the tasks Questionnaire (*Q*), Mathematics (*M*), and Genius (*G*); rows display frequency at Ap10, Ap20, and Ap30, as well as Duration and Amplitude. Each cell shows Spearman's *ρ* coefficient; the colour scale (light and symmetric) ranges from −1 (negative association) to +1 (positive association), shared across panels. Asterisks indicate significance (*p < 0.05; ^†^0.05–0.10). FFMQ scores were calculated using standardised reverse coding and prorating for missing items, in accordance with instrument guidelines.

Taken together, these results reinforce that dispositional mindfulness is associated with lower frequency (especially at Ap30) and shorter duration of episodes in AB individuals, whereas amplitude and the control group showed no meaningful associations (Figure 2).

## 4 Discussion

The present study demonstrated that the modulation of AB by cognitive tasks occurs differently between individuals with an AB diagnosis and controls. In the within‐group analyses, controls significantly increased the frequency of episodes at lower thresholds (Ap10) as cognitive demand increased, whereas the AB group already exhibited high frequency from the initial task and maintained this constant pattern across subsequent conditions. Analysis of the Δ(*Q*–*G*) contrast revealed that controls tended toward negative values—indicating a greater occurrence of episodes during Genius—while AB individuals presented values close to zero, reinforcing the persistence of the behaviour regardless of task complexity. At higher intensities (Ap20 and Ap30), the AB group consistently maintained higher frequencies across all conditions, with significant between‐group differences at these thresholds. These findings are consistent with the most recent international consensus, which conceptualises bruxism as a behaviour rather than a disorder, occurring along a continuum that may serve as either a risk or a protective factor depending on the context [1, 2]. This behavioural perspective helps interpret why AB individuals sustain high episode frequency independently of task complexity. Recent EMG studies further support this view by showing that contextual factors, such as social media use, may subtly modulate jaw motor activity but do not significantly alter the frequency or intensity of AB episodes under controlled laboratory conditions [41]. Importantly, recent evidence combining ecological momentary assessment (EMA) with EMG has provided standardised thresholds for identifying AB, suggesting that ≥ 3.2 events/h at 20% MVC with duration ≥ 1 s represents a reliable instrumental cutoff distinguishing AB from controls. These benchmarks strengthen the validity of using EMG‐based parameters in controlled paradigms, such as the present study, to capture persistent activity patterns in AB individuals [42].

This comparison highlights two distinct modulation patterns: controls exhibited an adaptive response, with increased frequency as cognitive load rose, whereas AB individuals showed a persistent and stable pattern independent of context. Similar results have been reported in patients with masticatory muscle pain, who displayed a significantly greater frequency of activity episodes than controls, without relevant task‐related variation [10, 42]. Consistently, cognitive and fine manual activities such as reading and computer use have been shown to increase AB episodes in young adults [11]. Among university students, AB prevalence has also been linked to higher levels of perceived stress, suggesting that contextual factors may potentiate the persistence of this behaviour [33]. From a neurofunctional perspective, tasks requiring sustained attention activate cortical areas involved in motor control, which may explain the increased frequency observed in controls under greater cognitive load [43]. Moreover, recent ecological studies emphasise that AB metrics are sensitive to contextual modulation, with frequency being particularly responsive to stress and attentional demands [6]. Complementing this, longitudinal EMG monitoring demonstrated that day‐to‐day variation in masseteric waveforms is relatively low, supporting the robustness of frequency as a stable marker for differentiating between AB and non‐AB individuals [37].

Studies employing divided‐attention tasks have demonstrated that cognitive overload modulates motor preparation, reflected in reduced amplitude of movement‐related cortical potentials (MRCP) under dual‐task conditions [43]. Neuromodulation strategies further indicate that stimulation of cortical regions involved in motor and attentional control, such as the primary motor cortex and dorsolateral prefrontal cortex, can modify aspects of attention and working memory, particularly in high‐demand conditions [44, 45]. Complementarily, non‐invasive cognitive interventions such as brief mindfulness training have been effective in improving working memory, enhancing sustained attention, and reducing fatigue [22], suggesting that distinct approaches—whether neurostimulation or attentional training—converge on the modulation of critical cognitive resources underpinning motor responses. Nevertheless, recent EMG investigations have highlighted that short‐term laboratory tasks may underestimate variability in AB behaviours, stressing the importance of ambulatory recordings and ecologically valid conditions. Such evidence indicates that laboratory paradigms should be complemented with real‐life assessment tools to capture the full spectrum of AB activity [36, 38].

Regarding the duration and amplitude of AB episodes, no significant differences were identified between tasks or groups, except for a slight trend toward greater amplitude during Genius in controls. These findings suggest that frequency is a more sensitive metric to differentiate groups and experimental conditions, whereas duration and amplitude exhibit greater variability and lower stability. This interpretation is supported by previous investigations showing frequency as a more reliable marker [11, 42] and by the observation that amplitude tends to remain unstable even under baseline conditions [46]. This observation also resonates with the development of the BruxScreen, which focuses on pragmatic identification of AB‐related behaviours rather than rigid cut‐offs, acknowledging that variability in amplitude and duration limits their diagnostic sensitivity [34]. In addition, recent EMG‐based studies confirmed that parameters such as duration and amplitude show limited sensitivity in laboratory contexts, with no significant differences detected in tasks involving social media use, further reinforcing the superiority of frequency for AB assessment [42]. Conceptually, this aligns with updated recommendations that emphasise the clinical utility of frequency as the primary AB metric, given its higher reproducibility and ecological validity compared with other parameters.

Another relevant finding was the association between higher dispositional mindfulness and lower frequency and duration of episodes in the AB group, particularly at higher intensity thresholds. This pattern was not observed among controls, which is consistent with the hypothesis that mindfulness may be related to a protective effect specifically in individuals with AB. Our results align with recent evidence indicating that higher mindfulness levels are associated with lower AB frequency in real‐time ecological monitoring [9]. Clinical trials reinforce this potential, demonstrating that mindfulness‐based interventions reduce pain and psychological symptoms in patients with TMD [26] and improve bruxism symptoms in children when combined with sleep hygiene [4]. Conceptually, the use of the FFMQ allows exploration of specific facets—such as acting with awareness and non‐reactivity—that are related to reductions in automatic behaviours [23, 24]. Complementary neuroimaging studies indicate that mindfulness decreases activity in cortical networks associated with affective processing and enhances recruitment of cognitive control regions, suggesting specific neural mechanisms for attenuating repetitive behavioural patterns [15, 22]. These findings are corroborated by cross‐sectional evidence in the field of pain related‐TMD, which demonstrates that mindfulness buffers the painful experience, thus mitigating the vulnerability related to the cognitive‐behavioural‐emotional axis, that is also present in AB [25].

Overall, our findings reinforce that AB should be understood as a multifactorial behaviour modulated by psychological and contextual factors. The persistence of elevated frequency in AB individuals, independent of task demands, suggests dispositional components that go beyond a simple response to cognitive load. Epidemiological studies support this interpretation: in university students, AB has been associated with orofacial pain and higher perceived stress [33], with Brazilian prevalence rates ranging from 30% to 36% and strong links to academic overload [47]. From a global perspective, a recent meta‐analysis estimated an overall prevalence of ~23%, with higher rates in South America, highlighting the influence of regional and cultural factors [48]. Recent studies have also shown that AB is directly associated with temporomandibular disorders and elevated stress levels, underscoring the importance of interpreting AB within a biopsychosocial framework [49, 50, 51]. Accordingly, our findings align with the literature in indicating that AB cannot be explained solely by biomechanical factors but must be understood as the result of interactions among individual predisposition, environmental demands, and psychological regulation. The most recent consensus documents stress this same perspective, emphasising that AB metrics should be interpreted along a behavioural continuum, where frequency may serve as a marker of increased vulnerability to psychosocial influences [1, 2]. In this context, the proposal of evidence‐based EMG cut‐offs for AB detection provides an important step toward operationalising this continuum into clinically applicable thresholds, bridging research and practice [42].

Building on the modern behavioural definition of bruxism as a motor activity existing along a continuum, the critical shift from an adaptive physiological response to a maladaptive condition occurs when its frequency, intensity, and/or duration contribute to negative clinical consequences, such as orofacial pain and temporomandibular disorders (TMDs) (*Main document_rafa_revision1.docx, Discussion, References* [33, 49, 50, 51]). While low‐level masticatory muscle activity can be an adaptive response to cognitive or emotional demands, its persistent elevation, particularly under cognitive load as observed in our AB group, indicates increased vulnerability. This transition from adaptive to maladaptive is increasingly operationalised by objective EMG cut‐offs, with some evidence suggesting criteria like ≥ 3.2 events/h at 20% MVC with ≥ 1 s duration as reliable instrumental thresholds to delineate clinically significant AB [41]. Our findings, reinforcing the pattern of stable, elevated activity in AB individuals and the protective role of dispositional mindfulness, underscore the necessity of a multi‐dimensional assessment within a biopsychosocial framework to understand this adaptive‐maladaptive spectrum.

Some limitations should be acknowledged. First, the sample comprised young adults, restricting generalizability to other age groups. Furthermore, classification of participants as AB‐positive was based on short‐term clinical and instrumental criteria without longitudinal follow‐up, which may not fully capture temporal variability of the behaviour—a limitation also noted in previous cross‐sectional studies [9]. Another limitation concerns the EMG thresholds used (Ap10, Ap20, Ap30), which still lack universal standardisation, making comparisons across investigations challenging, as highlighted in recent analyses [6, 42]. Finally, the cross‐sectional design and use of standardised cognitive tasks in a laboratory setting, while allowing for greater experimental control, do not fully reproduce real‐life demands, potentially under‐ or overestimating the actual occurrence of episodes.

Furthermore, our cross‐sectional, laboratory‐based design did not allow for explicit control or real‐time monitoring of important covariates such as daily stress levels, sleep quality, and dynamic emotional states. While recognised as crucial modulators of bruxism activity and mindfulness levels in the literature [6, 8, 13], the integration of these complex variables was beyond the scope of this controlled experimental study, which focused on the effects of standardised cognitive tasks. This absence limits the ecological validity of our findings, suggesting that future research should incorporate longitudinal and ecologically valid assessments to capture the full, multifactorial interplay of these elements in real‐life contexts.

From a clinical perspective, the finding that AB individuals maintain a high and persistent frequency of episodes, whereas controls modulate behaviour according to cognitive load, underscores the need for diagnostic approaches that combine objective methods, such as electromyography, with real‐time monitoring tools. Recent evidence shows that ecological momentary assessment (EMA) captures substantial differences between patients with masticatory pain and healthy controls, providing more sensitive data on the impact of attentional and contextual factors [52]. In addition, the negative association between dispositional mindfulness and episode frequency observed in this study is consistent with findings identifying mindfulness as a protective marker against AB [9]. Clinical trials further suggest that mindfulness‐based interventions may reduce pain and improve psychological symptoms in patients with TMD, reinforcing their potential as a complementary resource in the clinical management of individuals with persistent clenching patterns [26]. Notably, randomised trials indicate that mindfulness‐based protocols not only alleviate psychological burden but also contribute to functional improvements in patients with chronic orofacial pain, providing translational relevance to our findings [26].

Looking ahead, the integration of digital technologies and behavioural strategies emerges as a promising avenue. Mobile‐based interventions have already demonstrated effectiveness in monitoring AB episodes in real time and differentiating patterns between patients with masticatory pain and controls [52]. This approach provides immediate feedback, enhances patient awareness, and facilitates interruption of automatic habits, particularly when combined with dispositional mindfulness, whose protective association has recently been evidenced in ecological monitoring [9]. The combination of mobile applications and mindfulness practices may offer personalised and accessible solutions, including for paediatric populations, as illustrated by reductions in bruxism among children undergoing brief guided meditation protocols [4]. Accordingly, there is a clear need for multidisciplinary strategies that address not only occlusal aspects but also psychological and contextual dimensions. Longitudinal studies with larger and more heterogeneous samples will be essential to validate these interventions and to understand their long‐term effects, particularly in preventing progression to orofacial pain and temporomandibular disorders.

This study sought to investigate the relationship between AB, standardised cognitive tasks, and dispositional mindfulness levels. The results partially confirmed the hypotheses: in controls, episode frequency varied according to cognitive load, consistent with prior investigations showing increased activity during reading or computer use [10, 11]. In AB individuals, however, behaviour remained elevated and stable regardless of task, a pattern similar to that observed in patients with masticatory pain [52] and in students under academic stress [33]. Contrary to expectations, duration and amplitude did not vary significantly, suggesting lower sensitivity of these metrics in short‐term experimental settings. In line with the second hypothesis, higher dispositional mindfulness levels were associated with lower frequency and duration of episodes, particularly at higher thresholds, consistent with recent evidence from ecological monitoring [9]. Clinical trials have also demonstrated the benefits of mindfulness in populations with orofacial pain and bruxism [4, 26], reinforcing the translational plausibility of our results. Thus, by highlighting the interaction between cognitive load, mindfulness, and EMG outcomes, this study contributes to diagnostic refinement and points toward novel therapeutic perspectives based on digital monitoring and behavioural intervention.

This study presents some limitations that should be considered when interpreting the findings. First, the sample consisted exclusively of young university students, which restricts the generalizability of the results to other age groups and clinical populations. In addition, although the diagnosis of probable awake bruxism was based on a combination of self‐report, clinical signs, and short‐term electromyographic criteria, this approach is more susceptible to misclassification compared with ecological momentary assessment or polysomnographic validation, potentially leading to under‐ or overestimation of prevalence. Another limitation concerns the cross‐sectional design, which precludes conclusions about causal or directional relationships. It remains unclear whether higher mindfulness levels actively reduce the occurrence of awake bruxism episodes or whether individuals with lower bruxism activity tend to report greater dispositional mindfulness. The relatively modest sample size also limited the statistical power to explore subgroup effects, such as sex differences or the contribution of specific mindfulness facets, which may provide more nuanced insights in future studies. Finally, the use of standardised laboratory tasks (mathematical exercises and electronic memory game) ensured experimental control but may not fully capture the complexity of real‐life situations in which awake bruxism typically occurs, such as prolonged screen exposure, academic overload, or social stressors. This limits the ecological validity of the results and suggests the need for future studies combining laboratory paradigms with naturalistic monitoring methods.

## 5 Conclusions

In conclusion, this exploratory cross‐sectional study demonstrated that controls tend to increase the frequency of masticatory muscle contraction episodes during cognitively demanding tasks, whereas individuals with probable AB maintain a higher and relatively constant frequency across standardised mental tasks of varying complexity. Furthermore, higher levels of dispositional mindfulness (FFMQ) were associated, within the AB group, with lower frequency—particularly at Ap30 (and to a lesser extent at Ap20)—as well as shorter episode duration across all tasks. Among controls, these associations were weak or absent. These findings do not imply causality and should be interpreted considering important limitations, including the cross‐sectional design, sample size, classification of AB as ‘probable,’ and the operationalization of EMG thresholds (Ap10/Ap20/Ap30). Therefore, they represent preliminary, hypothesis‐generating evidence that warrants replication in larger samples and longitudinal or experimental designs to clarify the underlying mechanisms of the observed effects.

## Author Contributions

R.C.M.‐F., C.S.P.S.: conceptualization, methodology, data curation, formal analysis, investigation, writing – original draft, project administration. R.C.M.‐F., C.S.P.S., N.J.S.C., F.‐C.L.‐O.: data curation, formal analysis, investigation, writing – review and editing. M.O.M., F.C.L.‐O., S.C.H.R., L.V.M., J.F.M.‐C.: data curation, visualisation, writing – review and editing. M.O.M., F.C.L.‐O., L.V.M., J.F.M.‐C.: conceptualization, methodology, visualisation, investigation, writing – review and editing, supervision, project administration.

## Ethics Statement

The present study was submitted to the Research Ethics Committee of the School of Dentistry of Ribeirão Preto, University of São Paulo, for evaluation and approval prior to the experimental stages (CAAE n°: 76595123.3.0000.5419).

## Conflicts of Interest

The authors declare no conflicts of interest.

## Data Availability

The data that support the findings of this study are available from the corresponding author, upon reasonable request.
